# On the genetic involvement of apoptosis-related genes in Crohn's disease as revealed by an extended association screen using 245 markers: no evidence for new predisposing factors

**DOI:** 10.1186/1477-5751-4-8

**Published:** 2005-11-30

**Authors:** Sonja EN Wagenleiter, Peter Jagiello, Denis A Akkad, Larissa Arning, Thomas Griga, Wolfram Klein, Jörg T Epplen

**Affiliations:** 1 Department of Human Genetics, Ruhr-University, Bochum, Germany; 2 Institute for Clinical Molecular Biology, University Schleswig-Holstein, Kiel, Germany; 3 Department of Gastroenterology, University Hospital Bergmannsheil, Bochum, Germany

## Abstract

Crohn's disease (CD) presents as an inflammatory barrier disease with characteristic destructive processes in the intestinal wall. Although the pathomechanisms of CD are still not exactly understood, there is evidence that, in addition to *e.g*. bacterial colonisation, genetic predisposition contributes to the development of CD. In order to search for predisposing genetic factors we scrutinised 245 microsatellite markers in a population-based linkage mapping study. These microsatellites cover gene loci the encoded protein of which take part in the regulation of apoptosis and (innate) immune processes. Respective loci contribute to the activation/suppression of apoptosis, are involved in signal transduction and cell cycle regulators or they belong to the tumor necrosis factor superfamily, caspase related genes or the BCL2 family. Furthermore, several cytokines as well as chemokines were included. The approach is based on three steps: analyzing pooled DNAs of patients and controls, verification of significantly differing microsatellite markers by genotyping individual DNA samples and, finally, additional reinvestigation of the respective gene in the region covered by the associated microsatellite by analysing single-nucleotide polymorphisms (SNPs). Using this step-wise process we were unable to demonstrate evidence for genetic predisposition of the chosen apoptosis- and immunity-related genes with respect to susceptibility for CD.

## Introduction

Crohn's disease (CD) is a chronic inflammatory disorder characterized by destructive processes in the intestinal wall. Interactions between genetic and environmental factors potentially lead to an imbalance between the luminal bacterial flora, and the innate as well as the adaptive immune systems [1,2]. Epidemiological and genome wide studies have lead to the identification of factors establishing genetic involvement in CD [1,3,4]. Despite of fundamental findings, namely the variation in the CARD15 receptor and their association with CD, the causative instances regulating the exaggerated mucosal response remained elusive. The proposed pathomechanisms of CD are manifold. The dysregulated response of the innate immune system is supposed to present a crucial step in the pathogenesis of CD [5]. This fact has been confirmed genetically by several CD associations of genes such as CD14, TLR4 and in some instances the interaction of their variations with CARD15 [6,7]. In regard to the polarized T helper (Th) response, the adaptive immune system appears affected in CD as well [8-10]. Moreover, several studies implicated a role of programmed cell death in CD [11-15]. Apoptosis mediates 'self-tolerance', the elimination of autoreactive immune compartments. In addition, the thoroughly controlled termination of a physiological immune response is due to the process of programmed cell death. In CD mucosal T cells show less susceptibility to apoptosis [16]. In this context TNFα protein exerts multiple physiological effects, and anti-TNFα therapeutic strategies (*e.g*. infliximab) are effective in (maintaining) remission of CD [17]. In several studies it has been revealed that treatment of CD patients with infliximab leads to an activation of T cells rendering them susceptible for apoptosis [18,19]. Interestingly, the effect of this treatment may not be due to neutralisation of soluble TNFα (and its binding to the TNFRs), but rather it may be caused by its affinity to membrane-bound TNFα putatively changing the ratio of anti- and pro-apoptotic mediators towards induction of apoptosis [18,20]. Although the mechanisms of the causal role of T cells responses in CD remain to be determined in detail, there is substantial clinical evidence that suggests a role for uncontrolled activated T lymphocytes in the pathogenic process of CD [21-24]. Nevertheless, it is uncertain, whether a genetic basis for a decreased activation/apoptosis of T lymphocytes in CD patients exists, and whether increased anti-apoptotic markers, found in T cells of these patients are due to the mucosal inflammation, secondarily [18].

In such a complex situation we used extended association screening (EAS) with markers representing 245 apoptosis- and (innate) immunity-related genes. The majority of the investigated markers have been successfully utilized in respective studies before [25,26]. Our population based linkage mapping comprises a 3-stage analysis with pooled DNA in the initial phase and subsequently individual genotyping. In order to confirm such results, several tagging SNPs of the adjacent gene represented by the marker were analysed. Here, we investigated the role of distinct biological pathways for the susceptibility of CD.

## Materials and methods

### Patients

One hundred and fifty eight well-characterized patients with a clinical, endoscopical and histological diagnosis of CD were included. This patient cohort has been reported before [27,28]. All patients were of German origin and the diagnosis of CD was adjusted according to the diagnostic criteria of the European Community Workshop on Inflammatory Bowel Diseases (IBD). As controls a group of healthy northern German (NoG) and western German (WeG) origin were analysed. In the initial step a group of ~100 NoG individuals were used. In order to exclude population stratification, genotyping of chosen SNPs was performed in 180–460 NoG and WeG individuals.

### Pooling of DNA

The DNA concentration from each individual of the patient and control cohorts was quantified by spectrophotometry, carried out four times, and then diluted accordingly to 100 ng/μl. In a second step the DNA was diluted to a concentration of 65 ng/μl and once more measured by spectrophotometry. Finally, DNA diluted to 50 ng/μl was adjusted to a final amount of 1000 ng for each individual in a pool of 50 persons. In the initial stage, marker analyses were performed with two patient and two control subpools, respectively.

### Tailed primer PCR

Tailed primer PCR was performed as described before [25]: An 18 bp-tail was added to each sense oligonucleotide. PCR reaction included three oligonulceotides, two of which were target specific. The third one consists of the same sequence as the abovementioned tail that was additionally fluorescence-labelled.

### Microsatellite markers

Intragenic microsatellite or markers located in the immediate vicinity (<50 kb) of the specific gene were included. Information on the oligonucleotide sequences and location of markers are given at the website (Additional file 1; see also Tab. 1). As reported before, only markers with equal "intra-subgroup" allele distributions with ≥ 2 alleles were considered in subsequent analyses [25]. Significantly associated markers were genotyped individually in order to exclude false-positive results due to possible pooling artefacts. All in all, 245 microsatellite markers representing distinct genes were analysed on an ABI377 slab-gel system (Applied Biosystems, Darmstadt, Germany).

**Table 1:** Genes investigated for CD association as represented by an intra- or juxtagenic microsatellite marker (for additional information see URL: )

**apoptosis related**	*REQ*	*TNFSF12*	*CTLA4*	*Casp10*	*IL4*
	*RNF7*	*TNFSF14*	*DAP*	*Casp14*	*IL4R*
	*SMAC*	*TNFSF15*	*DAPK1*	*Casp2*	*IL6*
*AIF*	*TIAF1*	*TNFSF18*	*FADD*	*Casp3*	*IL8*
*APR3*	*TIAL1*	*TNFSF4*	*IKBKG*	*Casp4*	*IRF1*
*BCLG*	*TP73*	*TNFSF5*	*MADD*	*Casp5*	*NRG1B*
*BFAR*	*VDR*	*TNFSF6*	*MAP2K6*	*Casp6*	*PRL*
*CIDEB*		*TNFSF7*	*MAP3K14*	*Casp7*	*PRLR*
*CYBB*	**Bcl2 related**	*TNFSF8*	*MAP3K5*	*Casp8*	
*CYP51*		*TNFSF9*	*MAP4K4*	*CASP8AP2*	**chromosome 6**
*DAD1*	*BCL2A1*	*TOSO*	*NFKB1*	*Casp9*	
*DAP3*	*Bag1*		*NFKB2*		*No.1*
*DATF1*	*BAK*	**innate immunity**	*NSMAF*	**apoptosis suppressor**	*No.4*
*DAXX*	*BAX*		*PAWR*		*No.5*
*DEDD*	*BCL2*	BPI	*PIAS3*		*No.6*
*DHCR24*	*BCL2L1*	*CD14*	*PTEN*	*API5*	*No.7*
*EIF4G2*	*BCL2L11*	*CD5L*	*RARB*	*BIRC1*	*No.8*
*FASTK*	*BCL2L13*	*DEFB119/ DEFB121*	*RIPK1*	*BIRC2*	*D6S1014*
*FLIP*	*BID*	*DEFB127*	*RIPK2*	*BIRC3*	*D6S1959*
*FRZB*	*BIK*	*HBD1*	*RIPK3*	*BIRC4*	*D6S273*
*GSK3B*	*BNIP3L*	*IFNB1*	*RXRB*	*BIRC6*	
*GSR*	*MCL1*	*LY64*	*STK17A*	*BIRC8*	**others**
*GZMA*		*LY86*	*STK17B*		
*GZMB*	**TNF superfamily**	*LY96*	*TANK*	**cytokine chemokines**	*BPHL/TUBB*
*HLCS*		*NCF1*	*TRADD*		*TAPBPR*
*NME3*	*LTB (TNFSF3)*	*NCF4*	*Traf3*		*VEGF*
*NOL3*	*LTBR (TNFRSF3)*	*PGLYRP*	*Traf4*	*AXL*	*LGALS3*
*NOS1*	*TNFa*	*PLA2G4A*	*Traf5*	*CSF1R*	*BDNF*
*NOS2A*	*TNFRSF10A*	*PLUNC*	*Traf6*	*CSF2*	*NGFB*
*NOX1*	*TNFRSF10B*	*SerpinA1*		*CSF2RB*	*NGFR*
*NOX3*	*TNFRSF10C*	*SerpinB1*	**cell cycle regulators**	*CSF3*	*TrkC*
*NOX4*	*TNFRSF10D*	*SFTPA1*		*Dtk*	
*P2RX1*	*TNFRSF11A*	*SLPI*	*CCND2*	*erbB3*	**positive control**
*P53AIP1*	*TNFRSF11B*	*STAT3*	*CDC2*	*GAS1*	*CARD15*
*PDCD10*	*TNFRSF12*	*TGFB1*	*CDKN1A*	*IGF1*	
*PDCD2*	*TNFRSF17*	*TLR1*	*CDKN2A*	*IGF2R*	
*PDCD5*	*TNFRSF18*	*TLR2*	*PAK1B*	*IL10*	
*PDCD6*	*TNFRSF19*	*TLR3*	*RbAp48*	*IL10RA*	
*PDCD6IP*	*TNFRSF19L*	*TLR4*	*Rb2/p130*	*IL10RB*	
*PDCD8*	*TNFRSF1A*	*TLR5*	*RBP1*	*IL11RA*	
*PLA2G10*	*TNFRSF1B*	*TLR7*	*RBP2*	*IL12A*	
*PLA2G1B*	*TNFRSF21*	*TLR8*	*RBQ-1*	*IL12B*	
*PLA2G6*	*TNFRSF4*	*TLR9*	*RBQ-3*	*IL12RB2*	
*PTGS1*	*TNFRSF5*	*TLR10*	*TP53*	*IL13RA2*	
*REQ*	*TNFRSF6 (FAS)*		*TP53INP1*	*IL18*	
*RNF7*	*TNFRSF6B*	**signal transduction**		*IL18R*	
*SMAC*	*TNFRSF7*		**caspase related**	*IL1RL1*	
*TIAF1*	*TNFRSF8*	*Traf1*		*IL1B*	
*TIAL1*	*TNFRSF9*	*BCL10*	*ADPRT*	*IL2*	
*TP73*	*TNFSF10*	*CHUK*	*CARD4*	*IL24*	
*VDR*	*TNFSF11*	*CRADD*	*Casp1*	*IL2RA*	

### Statistics for initial comparisons of allele frequencies

Raw data from ABI377 profiles were analysed by the Genotyper software (ABI) producing a marker specific allele image profile (AIP) which includes different heights of peaks reflecting the allele frequencies. In order to test differences of the AIPs between CD patients and the controls, all peak heights were summarized for each pool and set to 100 %. The total allele count for each distinct allele was then estimated. Thereupon, the AIPs of the case and control pools were compared statistically by means of contingency tables. Hence, P values are nominal and approximate, because estimated rather than observed counts were used for allele frequencies. The significance level was set at p = 0.05. In order to focus the statistics on major alleles, all minor alleles with a frequency of less than 0.05 were summarized to a virtual allele. Subsequently, a second statistical analysis by means of contingency tables was undertaken. A third step for statistical testing each allele individually was accomplished (and the summation of all other marker alleles), whereby the respective value of the patient group was compared with those of the controls and subsequent χ^2 ^analyses. Despite of evidence that correction for multiple comparisons might eliminate 'real positive' results [26], Q value correction was performed with a cut off of 5% for the initial screening procedure [29].

Nevertheless, for selecting markers for further investigations, non-corrected P values were simply ranked according to their evidence for association including all performed statistical procedures.

### Individual genotyping

Markers with significantly different allele distributions between patients and controls were controlled by genotyping individual DNA samples of patients and controls in order to exclude false-positive results due to pooling artefacts. Individual genotyping was performed by capillary gel electrophoresis by using the BeckmanCoulter CEQ8000 genetic analysis system (Beckman Coulter, Germany). Results were analysed by comparing each microsatellite allele frequency from the CD cohort with the corresponding allele frequency of the control group by χ^2 ^testing and corrected by the number of marker specific alleles according to Bonferroni (see Tab. 2 and URL: ). Hardy-Weinberg equilibrium (HWE) was tested using the Genepop program .

**Table 2:** P values for microsatellite markers located intragenically or in the immediate vicinity of represented genes after the initial step and individual genotyping.

	**p values**				
	
**gene (as represented by the respective marker)**	**after analysis with pooled DNA**	**after summation of alleles beneath 5%**	**after analyses of each single allele (most significant allele)**	**after individual genotyping^1 ^(p^c ^value)**	**after correction by Q-value of pooled data**
*FLIP*	0.2871	0.1936	0.0100	0.0044 (p^c ^> 0.05; c = 9)	n.s.
*BCL2A1*	0.0948	0.0948	0.0275	n.s.	n.s.
*BAG1*	0.2541	0.2541	0.0163	n.s.	n.s.
*BPI*	0.0011	0.0011	0.0031	n.s.	n.s.
*erbB3*	0.0760	0.0932	0.0100	n.s.	n.s.
*TP73*	0.5928	0.3535	0.0302	n.s.	n.s.
*TLR9*	0.3004	0.3004	0.0300	n.s.	n.s.
*TNFRSF17*	0.0012	0.0014	0.0014	0.0012 (p^c ^< 0.01; c = 6)	n.s.
*CARD15*	0.0083	0.0247	0.0054	0.0050 (p^c ^< 0.04; c = 7)	n.s.

P values were generated using three different procedures as described in the methods' section. Briefly, data were analysed by means of contingency tables, initially comparing allele distributions represented by the AIF (after analyses with pooled DNA), then after summation of alleles < 5% in order to focus on the major alleles and, finally, after comparison of each single allele between the control and patient cohorts. For analysing the results of the individual genotyping χ^2 ^testing was utilised.

^1^Genotyping was performed with the same individuals used in the pooling procedure, and, when remaining significant, further individuals were added to the analyses (FLIP: CD = 134, controls = 150; *TNFRSF17*: CD = 147, controls = 135; *CARD15*: CD = 144, controls = 165).

### SNP genotyping

SNPs in genes as represented by significantly associated markers after individual genotyping were investigated by analysis of restriction fragment length polymorphisms (RFLP; see Tab. 3). As the marker representing the *TNFRSF17 *gene is located in ~1 MBp distance to the *MHC class II transactivator *(*MHC2TA*) gene, a functional variation (rs3087456, [30]) of *MHC2TA *was genotyped by RFLP analyses in 147 CD patients and 463 healthy controls from the abovementioned control populations (see Tab. 3). The results were evaluated by means of χ^2 ^-and HWE testing. Linkage disequilibrium (LD) between the marker alleles and the polymorphism was calculated by the Genepop program.

**Table 3:** Investigated SNPs in genes as represented by significantly differing microsatellites of the individual genotyping step.

**Gene**	**rs#**	**Allele 01/02**	**Oligonucleotides (sense/antisense)**	**RE**	**TM (°C)**	**Allele: fragment length (bp)**
*FLIP*	Rs7583529	A/C	GGTGATTATTCGGACCCCA/AACTACAGATCCCGTGTGGAG	TseI	57	01: 155 02: 103/52
	Rs2041765	T/C	GAACAAGGAGAGAACCTGGAC/GAGCTGGAAGGCACAGTACA	MboII	56	01: 309 02: 188/121
*TNFRSF17*	Rs3743591	A/G	ATAAGCAGTTTCTGTTTCAGATGT/CTCTACAAGAATTCCAGAGCA	BceAI	55	01: 223 02: 147/76
	Rs11570139	C/T	GCCCTGATATTTACACCCTGT/CAGCCATCTGCAACATGAT	CaiI	54	01: 269 02: 161/108
	Rs373496	T/C	AGGAACTGAAACTCACAATAGC/CAGCTCATTATCTGTCTGATGTT	AluI	55	01: 247 02: 100/90/54/3
*MHC2TA*	Rs3087456	G/A	* ^1 ^GTGAAATTAATTTCAGAG**C**TGT/CTCAGCTTCCCCAAGGAT	BfmI	58	01: 268 02: 231/37

Analyses were performed by using the RFLP method. The table depicts information on the used SNPs as well as RFLP/PCR conditions. * ^1 ^A 5'-tail was added to the mismatch (bold letter) sense primer (5'-CATCGCTGATTCGCACAT-3'). PCR was performed with a third oligonucleotide with the equal sequence as the tail. RE: restriction enzyme; TM: melting temperature (used for annealing in PCR).

## Results

### Initial step

Microsatellites representing 245 genes involved in apoptosis regulation (see Tab. 1) were investigated by using EAS. None of the markers presented with significant intra-subgroup differences confirming the homogeneity of the pools. The statistical evaluation of the microsatellite frequencies in the CD patient and the control cohorts revealed 9 significantly different allele distributions of intra- or juxtagenic markers for *FLIP*, *BCL2A1*, *BAG1*, *BPI, erbB3*, *TP73*, *TLR9*, *TNFRSF17 *and *CARD15 *(summarized in Tab. 2).

### Individual genotyping

Individual genotyping confirmed significant P values only for the 3 markers *FLIP *(p = 0.0044, p_c _> 0.05, in HWE), *TNFRSF17 *(p = 0.0012, p_c _< 0.01, in HWE) and the positive control CARD15 (p = 0.0050, p_c _< 0.04, in HWE). The additional associations for the other markers were rejected (see Tab. 2 and Additional file 1). There were no differences analysing CARD15^+ ^and CARD15^- ^patients.

### SNP genotyping

SNP markers (Tab. 3) were genotyped located in the respective genes in the vicinity of the microsatellites representing *TNFRSF17 *and *FLIP*. Thus, SNPs were analyzed spread across the genes representing haplotypes as predisposed by the 'LD Select' method reported before [31]. RFLP analyses did not reveal any association of the selected SNPs, neither by comparing the CARD15^+ ^nor the CARD15^- ^patients with the control group.

### Comparison of *TNFRSF17 *microsatellite alleles

The genotypes of the *TNFRSF17 *microsatellite alleles were compared between the patient and control cohorts. Analyses revealed evidence either for a predisposing (allele 3) and a protective allele (2) or linkage between these alleles and the marker alleles, respectively.

**Figure 1:**
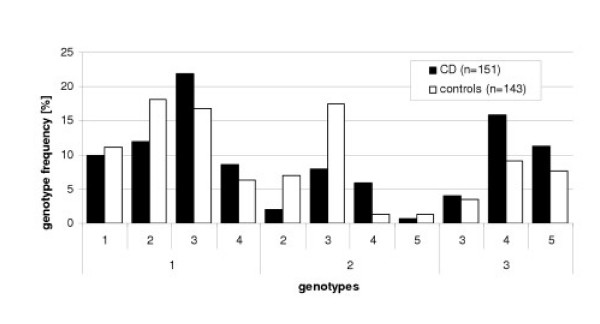
Genotype frequencies of the microsatellite representing the *TNFRSF17 *gene. Only genotypes with a frequency of > 0.01 are included. Alleles of the respective microsatellite are indicated as numbers in the X-axis according to their length in bp. For example: 1–1 (read from the number below the numerical series and the first number of the numerical series) means homozygous genotype for microsatellite allele number one and 1–4 heterozygous genotype for allele 1 and 4. Genotypes comprising allele 2 are over-represented within the control group (47% *vs*. 29%; p_c _= 0.0042 with c = 2), whereas allele 3 genotypes are more frequent in the patient cohort (58% *vs*. 52% p^c ^= 0.3130; c = 2). Therefore, allele 2 might imply a protective effect and/or allele 3 a predisposing effect on CD. Interestingly, the genotype 2–3 is more prevalent in the control group. This result can be interpreted by a different effect size of allele 2 (↑) as compared to allele 3, or the significant difference of this microsatellite is only due to linkage of allele 2 with a protective factor.

Genotypes including allele 2 are overrepresented in the control cohort, whereas those with the apparently predisposing allele 3 are more frequent in the CD cohort, thus confirming the results of individual genotyping (see Fig. 1).

### *MHC2TA *analyses

The analyses of the functionally significant polymorphism rs3087456 revealed a marginal association in our CD patients when allele or genotype frequencies were compared between the combined control (WeG and NoG did not differ in allele frequencies) and the patient cohorts (see Tab. 4). Analyses for LD between *TNFRSF17 *and *MHC2TA *alleles, however, did not reveal any significant deviations from equilibrium.

**Table 4:** Allele and genotype frequencies of the functional *MHC2TA *polymorphism (rs3087456).

	Allele frequencies		p value	OR (CI)	Genotype frequencies		p value
				
CD (n = 147)	C	0.32	0.05	1.33 (0.90–2.01)	CC	0.08	0.54
							
	T	0.68			CT	0.48	0.06
					TT	0.44	0.03
controls (n = 463)	C	0.26			CC	0.07	
	T	0.74			CT	0.39	
					TT	0.54	

OR: odds ratio; CI: 95% confidence interval

## Discussion

The pathomechanisms of CD are still not exactly understood, albeit certain *CARD15 *variations appear especially frequent in CD patients; thus genetic involvement is proven. These genetic predisposition factors, however, are neither sufficient nor explain they the pathogenesis in all CD patients. In this study we present an association screen mainly for apoptosis and immunity related genes by microsatellite markers as investigated in a 3-step approach.

Our initial analyses revealed 9 significantly different allele distributions of intra- or juxtagenic markers for *FLIP*, *BCL2A1*, *BAG1*, *BPI, erbB3*, *TP73*, *TLR9*, *TNFRSF17 *and *CARD15 *(see Tab. 2). Yet, after correction by Q-value, none of those markers remained significant. On the other hand, a recent study raised the question, whether the correction for multiple comparisons should be applied at all in EAS [26]. For example, in these analyses a previously significantly associated microsatellite (representing the *TNFα *gene), which has been used as a positive control such as *CARD15*, would have been rejected by the correction procedure. Therefore, it remains conceivable that the abovementioned markers represent rather hints for additional predisposing factors/loci with low effect size.

The most promising markers (reflected by a significant p-value) were included in further analyses regardless of the correction procedure. Individual genotyping rejected most markers found to be significantly different in the initial step of our approach and only three markers remained significant representing the *TNFRSF17*, *FLIP*, *CARD15 *genes (Tab. 2). Obviously, pooled and individual genotyping yield somewhat contradictory results. Eight microsatellites revealed significantly differences between the patient and control cohorts after the pooling procedure, whereas individual genotyping results in the confirmation of 'only' 2 markers. These conspicuous differences might be due to several artefacts caused by analyses with pooled DNA. For example, a typical artefact is the length-dependent amplification of short alleles or the presence of null-alleles. Additionally, consistency of the analyses by a slab-gel system might reflect a further hindrance in this subtle procedure. Nevertheless, individual genotyping eliminates false positive results due to pooling artefacts and, in case of significant results, enables the thorough analyses of the marker alleles in detail (see Fig.1). In order to confirm the aforementioned positive results further markers (SNPs, Tab. 3) were genotyped located in the respective genes in the vicinity of the microsatellites representing *TNFRSF17 *and *FLIP*. Yet, RFLP analyses did not reveal any association of the selected SNPs and, therefore, the microsatellite data were not confirmed. On the other hand, these SNPs might not represent regions properly that encompass regulatory elements.

In some instances, the LD of distinct microsatellite alleles covers long genetic distances, thus further gene variations might be in linkage with these alleles. Since the significantly associated '*TNFRSF17*' marker is located at the IBD8 region with 1MBp distance to the major histocompatibility class (MHC) II transactivator (*MHCIITA*), a previously reported functional variation of the *MHC2TA *gene was analysed (see Tab. 4; [30]). MHC2TA regulates the expression of *human leukocyte antigen *(*HLA*) genes regulating the adaptive immune system by presenting antigens to CD4+ T cells, thereby re-activating these cells. The *HLA *region has been implicated in IBD [32]. In addition to the localisation of *MHC2TA *at IBD8 and the associated marker in the adjacent region, the putative biological relevance of the functional rs3087456 polymorphism for CD motivated us to genotype this variation. The analyses did reveal a marginal association in our CD patients when allele or genotype frequencies were compared between the combined control and patient cohorts (see Tab. 4). Yet there was no evidence for LD between *TNFRSF17 *and *MHC2TA *alleles. In order to validate these data further patient cohorts comprising more individuals must be scrutinised. In addition, other genes that might be linked with the '*TNFRSF17*' marker must be analysed (at least 15 RefSeq genes in the region are encompassed by the microsatellite marker and *MHC2TA*).

In conclusion, this study did not reveal overt evidence for CD predisposition factors in apoptotic (and immune) pathways. Certainly, our approach depends on the LD between the investigated microsatellites and putative predisposing or protective alleles, depending on functional relevance to the disease. Thus, in some instances microsatellites might not be entirely representative for the adjacent genes. Furthermore, the investigated genes only cover part of the factors which coordinate programmed cell death. Yet, future information about haplotype blocks may facilitate more far-fetched interpretations of our analyses.

## Supplementary Material

Additional File 1 This file provides detailed information on the sequence of used oligonucleotides, represented gene, marker distance to gene and kind of nucleotide repeat (di, tri, etc.). Furthermore, the file includes graphical information on individually genotyped microsatellites markers with significant differences in allele distributions. Click here for file

## References

[B1] Russel MG, Pastoor CJ, Janssen KM, van Deursen CT, Muris JW, van Wijlick EH, Stockbrugger RW (1997). Familial aggregation of inflammatory bowel disease: a population-based study in South Limburg, The Netherlands. The South Limburg IBD Study Group. Scand J Gastroenterol Suppl.

[B2] Hugot JP, Thomas G (1998). Genome-wide scanning in inflammatory bowel diseases. Dig Dis.

[B3] Hugot JP, Chamaillard M, Zouali H, Lesage S, Cezard JP, Belaiche J, Almer S, Tysk C, O'Morain CA, Gassull M (2001). Association of NOD2 leucine-rich repeat variants with susceptibility to Crohn's disease. Nature.

[B4] Ogura Y, Bonen DK, Inohara N, Nicolae DL, Chen FF, Ramos R, Britton H, Moran T, Karaliuskas R, Duerr RH (2001). A frameshift mutation in NOD2 associated with susceptibility to Crohn's disease. Nature.

[B5] Rogler G (2004). Update in inflammatory bowel disease pathogenesis. Curr Opin Gastroenterol.

[B6] Franchimont D, Vermeire S, El Housni H, Pierik M, Van Steen K, Gustot T, Quertinmont E, Abramowicz M, Van Gossum A, Deviere J (2004). Deficient host-bacteria interactions in inflammatory bowel disease? The toll-like receptor (TLR)-4 Asp299gly polymorphism is associated with Crohn's disease and ulcerative colitis. Gut.

[B7] Klein W, Tromm A, Griga T, Folwaczny C, Hocke M, Eitner K, Marx M, Duerig N, Epplen JT (2003). Interaction of polymorphisms in the CARD15 and CD14 genes in patients with Crohn disease. Scand J Gastroenterol.

[B8] Pallone F, Blanco Gdel V, Vavassori P, Monteleone I, Fina D, Monteleone G (2003). Genetic and pathogenetic insights into inflammatory bowel disease. Curr Gastroenterol Rep.

[B9] Okazawa A, Kanai T, Watanabe M, Yamazaki M, Inoue N, Ikeda M, Kurimoto M, Ishii H, Hibi T (2002). Th1-mediated intestinal inflammation in Crohn's disease may be induced by activation of lamina propria lymphocytes through synergistic stimulation of interleukin-12 and interleukin-18 without T cell receptor engagement. Am J Gastroenterol.

[B10] Singh UP, Singh S, Iqbal N, Weaver CT, McGhee JR, Lillard JW (2003). IFN-gamma-inducible chemokines enhance adaptive immunity and colitis. J Interferon Cytokine Res.

[B11] Souza HS, Tortori CJ, Castelo-Branco MT, Carvalho AT, Margallo VS, Delgado CF, Dines I, Elia CC (2005). Apoptosis in the intestinal mucosa of patients with inflammatory bowel disease: evidence of altered expression of FasL and perforin cytotoxic pathways. Int J Colorectal Dis.

[B12] Xia B, Yu YH, Guo QS, Li XY, Jiang L, Li J (2005). Association of Fas-670 gene polymorphism with inflammatory bowel disease in Chinese patients. World J Gastroenterol.

[B13] Peppelenbosch MP, van Deventer SJ (2004). T cell apoptosis and inflammatory bowel disease. Gut.

[B14] Brannigan AE, O'Connell PR, Hurley H, O'Neill A, Brady HR, Fitzpatrick JM, Watson RW (2000). Neutrophil apoptosis is delayed in patients with inflammatory bowel disease. Shock.

[B15] Seidelin JB, Nielsen OH (2003). [Apoptosis in chronic inflammatory bowel disease. The importance for pathogenesis and treatment]. Ugeskr Laeger.

[B16] Ina K, Itoh J, Fukushima K, Kusugami K, Yamaguchi T, Kyokane K, Imada A, Binion DG, Musso A, West GA (1999). Resistance of Crohn's disease T cells to multiple apoptotic signals is associated with a Bcl-2/Bax mucosal imbalance. J Immunol.

[B17] Hommes DW, van Deventer SJ (2003). Targeting tumor necrosis factor-alpha in inflammatory bowel disease: why, how, and when?. Curr Opin Gastroenterol.

[B18] ten Hove T, van Montfrans C, Peppelenbosch MP, van Deventer SJ (2002). Infliximab treatment induces apoptosis of lamina propria T lymphocytes in Crohn's disease. Gut.

[B19] Van den Brande JM, Braat H, van den Brink GR, Versteeg HH, Bauer CA, Hoedemaeker I, van Montfrans C, Hommes DW, Peppelenbosch MP, van Deventer SJ (2003). Infliximab but not etanercept induces apoptosis in lamina propria T-lymphocytes from patients with Crohn's disease. Gastroenterology.

[B20] Sands BE (2004). Why Do Anti-Tumor Necrosis Factor Antibodies Work in Crohn's Disease?. Rev Gastroenterol Disord.

[B21] Sturm A, Leite AZ, Danese S, Krivacic KA, West GA, Mohr S, Jacobberger JW, Fiocchi C (2004). Divergent cell cycle kinetics underlie the distinct functional capacity of mucosal T cells in Crohn's disease and ulcerative colitis. Gut.

[B22] Sartor RB (1995). Current concepts of the etiology and pathogenesis of ulcerative colitis and Crohn's disease. Gastroenterol Clin North Am.

[B23] Doering J, Begue B, Lentze MJ, Rieux-Laucat F, Goulet O, Schmitz J, Cerf-Bensussan N, Ruemmele FM (2004). Induction of T lymphocyte apoptosis by sulphasalazine in patients with Crohn's disease. Gut.

[B24] Elson CO, Sartor RB, Tennyson GS, Riddell RH (1995). Experimental models of inflammatory bowel disease. Gastroenterology.

[B25] Jagiello P, Gencik M, Arning L, Wieczorek S, Kunstmann E, Csernok E, Gross WL, Epplen JT (2004). New genomic region for Wegener's granulomatosis as revealed by an extended association screen with 202 apoptosis-related genes. Hum Genet.

[B26] Wieczorek S, Jagiello P, Arning L, Dahmen N, Epplen JT (2004). Screening for candidate gene regions in narcolepsy using a microsatellite based approach and pooled DNA. J Mol Med.

[B27] Klein W, Tromm A, Griga T, Fricke H, Folwaczny C, Hocke M, Eitner K, Marx M, Duerig N, Epplen JT (2002). A polymorphism in the CD14 gene is associated with Crohn disease. Scand J Gastroenterol.

[B28] Klein W, Tromm A, Griga T, Fricke H, Folwaczny C, Hocke M, Eitner K, Marx M, Duerig N, Epplen JT (2001). Interleukin-4 and interleukin-4 receptor gene polymorphisms in inflammatory bowel diseases. Genes Immun.

[B29] Storey JD, Tibshirani R (2003). Statistical significance for genomewide studies. Proc Natl Acad Sci U S A.

[B30] Swanberg M, Lidman O, Padyukov L, Eriksson P, Akesson E, Jagodic M, Lobell A, Khademi M, Borjesson O, Lindgren CM (2005). MHC2TA is associated with differential MHC molecule expression and susceptibility to rheumatoid arthritis, multiple sclerosis and myocardial infarction. Nat Genet.

[B31] Carlson CS, Eberle MA, Rieder MJ, Yi Q, Kruglyak L, Nickerson DA (2004). Selecting a maximally informative set of single-nucleotide polymorphisms for association analyses using linkage disequilibrium. Am J Hum Genet.

[B32] Yap LM, Ahmad T, Jewell DP (2004). The contribution of HLA genes to IBD susceptibility and phenotype. Best Pract Res Clin Gastroenterol.

