# Supplementary material for: On the genetic involvement of apoptosis-related genes in Crohn's disease as revealed by an extended association screen using 245 markers: no evidence for new predisposing factors
Source: J Negat Results Biomed. 2005 Nov 30;4:8. doi: 10.1186/1477-5751-4-8 (PMC1315346; doi:10.1186/1477-5751-4-8)
Supplement: Additional File 1 — This file provides detailed information on the sequence of used oligonucleotides, represented gene, marker distance to gene and kind of nucleotide repeat (di, tri, etc.). Furthermore, the file includes graphical information on individually genotyped microsatellites markers with significant differences in allele distributions. [file 1477-5751-4-8-S1.pdf]

Information of used microsatellites in the EAS with apoptosis- and immune-related genes for MC

Sonja E.N. Wagenleiter *et al*. (2005)

represented gene	sense oligonucleotide	antisense oligonucleotide	marker distance to gene	*nucleotide marker
ADPRT	CGAAATTGTGGTAATGACTGC	AAGTCGAGGTGGGAGGATT	1kb	di (GT)
AIF	CCAGACCCTGTCTCTCAAAAT	CTCACCCCCACTAGTTCCA	intragenic	di (GT)
API5	TACAGGCATGCACTACCACA	TTGGAGTTTGAGACCAGCC	18kb	di (GT)
APR-3	AAACCATAGCACAGCCACATA	GTCTTAGCCCTGTGGTTTTGT	37kb	di (AC)
AXL	CTGCTTGTCCTAGCCTGTGT	TTCATTCCCCCTCACTCTCT	0.2kb	di (GT)
Bag1	AGGCAAAAAGTCGTGAATCA	CTGGTGACCTGAGAGAGCTG	28kb	di (CA)
BAK	TGCCTTCTTGGTGGTAGTGT	GCTCAGAAAGAGGAAGGGAC	7kb	di (AC)
BAX	GGGCAACAAGAGTGAAACTCT	CCTCAGTGCACAGCTCTACAT	19kb	di (AAG)
BCL10	GCACTGGGATTACAGGCAT	GCAAGCACAACCCAAACTATA	10kb	di (CA)
BCL2	GGTTCTCCTGACATGAGGC	AGGCAGGAGAATTGCTTGA	intragenic	tetra (ATTT)
BCL2A1	TCTGGGCAACAGAGTGAGAT	GGCTGGTCTCAAACTTCTGA	3kb	tri (AAT)
BCL2L1	CAAGACCGTGCCACTGTACT	TGGGATTAATTTGTTCGCC	intragenic	penta (AAAAT)
BCL2L11	ACTGCTGTCTTACGGACACAG	GTCAAGCCTCATGTTATGCTG	40kb	di (AC)
BCL2L13	CGATCATAGCTCAGTGCAGAC	AGGCAGGAGGATCATTTGA	intragenic	di (GT)
BCLG	AAGTCTTCCAAGCAAGCAGA	AAATCATCTTTTGGCTGGG	intragenic	di (AC)
BDNF	TCAAAAGTGTCACGTCATCCT	GCCTTCATGCTGAAGAGCT	intragenic	di (GT)
BFAR	AGTGCATAGTCTGGCTTCCTT	ACTTTGGGTCGAAGCAGTAAT	21kb	di (AC)
BID	CGATCATAGCTCAGTGCAGAC	AGGCAGGAGGATCATTTGA	13kb	di (GT)
BIK	GAGGCCAGGTGTTTGAGAC	CCCTGAGCTGTTCGTTCTT	35kb	di (AC)
BIRC1	GCATAAACATTCACAGCTTGC	GAGTTTCACTGGATGGAACG	intragenic	di (GT)
BIRC2	GTGTGGTTAGGTGCCATCAT	GAGTTTGAGACCAGCCTGG	intragenic	di (GT)
BIRC3	AAAAGACTGGGCTTGTCCTT	GCACATCTCTTACACCAGGG	intragenic	di (GT)
BIRC4	TCTGTGAGCTGAGATCATGCT	GTAGTGGGTAGCCCTGCTCT	intragenic	tetra (TAAA)
BIRC6	CATGACCTATTCCCGTGTTC	GGTACAGCAGCTCAGGAAACT	intragenic	di (GT)
BIRC8	CAGAATCTTTAATGGGCCTTT	ACACCATTGCACTCCAGTCT	1.5kb	di (AT)
BNIP3L	TTTTCGTTTATAGGGCCCA	CTGCACAGTCTCCCTCGTT	17kb	di (GT)
BPHL/TUBB	TTGTCACATGTCCGCTTTG	GACACATCAACGTGGCAGATA	0.7kb	di (GT)
BPI	CCAGACCACTCTCGCTGAA	AATAGTCGCAGCCCCTTCT	intragenic	di (AC)
CARD15	CATTGGTTTAAATTCCCTGGA	GCTGCACTGGATACTCCTGTT	7.9kb	di (AC)
CARD4	GACATCTGGCTCCAAAAGTAA	TGTGGCTATTGGAGGTTGATA	35kb	di (AT)
Casp1	CCCACAGCACATAGGGACT	TCCAACAAAGCATTGGATTC	21kb	di (GT)
Casp10	AAAATGCATGTCTGGCATG	TGCCCTGTGCTTCTATCTGTA	intragenic	di (GT)
Casp14	ATCATTTAAACCCAGGAGGC	GCAGGCAAACAAATCTTCAT	21kb	tetra (AAAT)
Casp2	CTCCATTTCTTCCTCTGCCT	CTTCAGTGAGCCGTGATCAT	6kb	tetra (TATT)
Casp3	GGCAACAAGAGCGAAACTC	CTTTACATTCCCCACAGTGC	8kb	tetra (AAAT)

represented gene	sense oligonucleotide	antisense oligonucleotide	marker distance to gene	*nucleotide marker
Casp4	TGAGGATATTTGGAGGACCA	TCTGACAGCCAGGGTAAGAA	intragenic	di (AC)
Casp5	CCCACAGCACATAGGGACT	TCCAACAAAGCATTGGATTC	intragenic	di (GT)
Casp6	GGCAGGACAACATACCTATCC	ACCCCTCAACATCAGTGATG	2.6kb	di (AT)
Casp7	GAGATGCAGACAGTTCCCAG	GGTCTGTCCCACCCTCTTAG	54kb	di (AT)
Casp8	CCTAATCCAGTATGGCCTCAT	AGCTTGGGCGATGTAGTGA	intragenic	di (GT)
CASP8AP2	AGGCTCAGGGAAGAGAATTG	ACACATGTCCAAGAGCAGGT	13kb	di (AC)
Casp9	GGCCCTCCAAATATATGGTT	TTCTGTAGAGCAATCTGGCAA	20kb	di (AC)
CCND2	GGAAAGGTTCAAGCAGCTCT	CCTCCCTCCAAACACTAATGT	intragenic	di (TG)
CD14	CAGGCCATCCGTTATTTCTT	CTTAGGACCCTGTGGCTTCT	14.7kb	di (GT)
CD5L	AAACCCCATAACCAATTCAGA	TCCCAGCCTCCATAATTGT	4kb	di (GT)
CDC2	TACGGCATAGCCTCCAGAT	GGAGCAAGAATGCCATTTAA	9kb	di (AC)
CDKN1A	TCACCACTGCGGTTTTACA	CTTAGGAGGCCCCAAACTT	15kb	di (GA)
CDKN2A	GGTTAGCAATAATTCTCCCCA	CAGAAGTCAGTGAGTCCCGA	intragenic	di (TG)
CHUK	ACGTCGGTTTTCATCTTGC	TTTCATGACATGGAAGCTAGC	intragenic	di (CT)
CIDEB	CAGAGCGAGATTCCACCTC	TCACTCATTTTTCACACGCA	10kb	tri (AAC)
CRADD	GGTACCATCAGCCTTCCAA	CCTTCTCTGCTTTGATTTCCT	intragenic	tri (TAT)
CSF1R	ATTTCCTGTGTCAGACCCTGT	ACC CTG TGT CTC AGT TTT CCT	intragenic	tetra (CTAT)
CSF2	TGGAGCAATTAGGAAAATGC	CAGTTGCCCATCTGGTAACTA	35kb	di (TG)
CSF2RB	AGACTGAGAGCTCCCTGAGC	GAACCAGCCAGCAAGAAAC	5kb	di (GT)
CSF3	CCAGCCCAGGAGTAGTCTTAC	CCAACAGCACACACAGTCAC	2.5kb	tetra (TAAA)
CTLA4	TGGCTATGTTTTAGCCAGTGA	ACGTGGCTCTATGCACAATAC	intragenic	di (AT)
CYBB	CCTATAATATTGTGCTTGCGC	CGTGATGACAACTCCAGTGAT	intragenic	di (TG)
CYP51	TTATCCATGGCCTTTCTTCTC	GCACTTTAGCCTTGGCAAC	intragenic	di (TG)
D6S1014	GGGTCTGACCACTGAGACAC	CAGTGAGAGCTCTGAGGGTC	Chro6 marker	tri (CAG)
D6S1959	CTGTGCCTCTATTGCCTCAT	TGAAGGCCAAGCACTATTTT	Chro6 marker	tetra (TAGA)
D6S273	GCAACTTTTCTGTCAATCCA	ACCAAACTTCAAATTTTCGG	Chro6 marker	di (GT)
DAD1	ACTATGATTCACGTCCAGCAG	CCCAGGGACCTTAAGTCATT	11kb	di (GT)
DAP	AGGAGGTGGAAGCAACTCA	GACGTGGCATATGACAGGAT	intragenic	di (AC)
DAP3	TGGAAACACTGTCCCCTGT	GCGTTCATGGATGTTTGTGT	29kb	di (AC)
DAPK1	GGAGGCTCCTACTCCTGTGTA	AGAACCTTCCAGGTGGTCATA	intragenic	di (AC)
DATF1	GGGAAAAGAAAATGGTGAGTC	GGCTGAGGTAGGAGAATCGT	33kb	tetra (TTTA)
DAXX	GGCAGGACAAAGTATTTTCCA	CACCGTATGTGTGAAGGTGAG	7kb	di (AC)
DEDD	GACACAGTTGACTTGAAGGCA	TTTTCTCTCCGCAGGACAC	8kb	tetra (TTTA)
DEFB119/DEFB121	TTTGTGCCTACTCCTCCCA	ATCCTGGCCTTCCTTTTCT	6.7kb / 7.5kb	di (AC)
DEFB127	TTCTGGGAAAGATGTGTTGTT	TGCTTCTTCCTCTATTCATGC	intragenic	di (TG)

represented gene	sense oligonucleotide	antisense oligonucleotide	marker distance to gene	*nucleotide marker
DHCR24	CATTGTGATGAGTCCAGCCT	AGCATTAAGGGAAAAGCATGT	40kb	di (AC)
Dtk	AGGAGAGGTGCGTGTATGTGT	AGCCACTGCCACATTAACAG	intragenic	di (GT)
EIF4G2	CTCCTTTTGCTGCATCTCATA	TGCAGTGAGCCAAGATCAC	7kb	di (TG)
erbB3	TGGGTGACACAGTGAGATCC	GATAGGCGCGGACTGTACA	intragenic	tetra (TTTC)
FADD	GGAGACAGAGTGAAACTCCGT	CTATGATGCCATTCACTGCA	7kb	tetra (AAAC)
FASTK	AGTCACTGCACTCCAGCCT	GAAGAGGGTCTGATTCACCAT	4kb	di (AC)
FLIP	AAGCCAAGATTGTGCTATTGC	CCATGTAGGGCCAACTAGAAT	intragenic	penta (TAAAA)
FRZB	GCCAAGGTCCTCTTGCTTA	AGGCTTTGCTCCAAAGTCTT	70kb	di (AC)
GAS1	CAATTGATCTGGGCCATGT	TCATCATGACAGCGTTGGT	8kb	di (AG)
GSK3B	ACTTGAGCCCGAAAAGTCA	TGTTGAACTTGGCATTCATCT	intragenic	di (AC)
GSR	AGA CGG AGG TTG CAG TGA G	CCT GTA CTA GGT CCC AGC AGA	intragenic	di (AC)
GZMA	TCC CAA CAC TCT CCT TTG TG	CCT TGG AGG CAT CCT TTA CT	5.6kb	di (CA)
GZMB	AACCAAAAACCACTTGTACCC	GTCCAGCAATCCCACTACTG	19kb	tetra (AAGC)
HBD1/DEFB1	GCCCAAAAAGAACAGCTAGAA	GTTCCTGCTTTTGTTTCGGT	intragenic	di (AC)
HLCS	GCCCATTTACCAGCTTCAA	TTATATAGCACATGCCTTGCC	intragenic	di (GT)
IFNB1	AAGTCGACACCCCAAATCA	CACCAAAGTGAGTGTGCCA	27.5kb	di (TG)
IGF1/IFNGR2	CCAACAAGCAAAACCATCAT	CTCTCCTGGACTGTGCACA	4kb	di (TG)
IGF2R	TGTTCAATTCATGTTGCTGC	CACAGCACTATCCATAATTGCA	intragenic	di (CA)
IKBKG	AGTGTGATCAGATCGGTTGG	CCTCCAGCTGTCAAAAGTCTT	intragenic	di (TG)
IL10	GCTGGATAGGAGGTCCCTTA	CTGGCTCCCCTTACCTTCTA	0.9kb	di (TG)
IL10RA	CAG CCT GGG AAA CAG AGT G	CCT GGA TTT CCC AGT CTC A	intragenic	di (AC)
IL10RB	CTGATCTATTGAGCCAGCCA	GGACAGGAGAGATCAAAGGAA	intragenic	di (TG)
IL11RA	CTTTTGTTCTGGGCCCTAA	ATACCCAGTGCTTTGCATGT	intragenic	di (CA)
IL12A	ATGGGATAAAAATATGGTGGC	TGGATGAAGCTCATTGCCT	intragenic	di (GT)
IL12B	CTGCCCTGTCATTTTCTCTCT	ACCATGCCCAGCATAGTTTAT	8kb	tetra (TATC)
IL12RB2	CTAACTGGTCTGCCTGCATC	GGGAAACCACTGGTGTTTAAT	intragenic	di (GT)
IL13RA2	CTCTCTCACCAGCTTTG C	CACGGAGCTTGACACCTAGTA	5.2kb	tri (ATA)
IL18	AATTCCAAGCTGTGAAGTGC	TCTTTCAACACATGGGCTTTA	9kb	di (TG)
IL18R	TGCCTTTCTGTTTCGTGTG	GAGAAAAGCCTTGCTCATCTT	4kb	di (GT)
IL1B	TCCTCCAAGAAATCAATCCAT	TCCACATCCCAAAAGTAATGA	17kb	tetra (ATCC)
IL1RL1	TGCCTTTCTGTTTCGTGTG	GAGAAAAGCCTTGCTCATCTT	intragenic	di (GT)
IL2	CGCTGCTGCTTATGATAGATC	TTGAGGCTCAATCTTGTTCCT	38kb	di (AT)
IL24	CCATCTGCTGAAGCCTAGAA	ATGCAGTGTGAGCTTTCAAAC	15kb	di (GT)
IL2RA	TGACCTCAGGTGATCTGCC	TTTGTAGCAAAGCAGCAATGT	intragenic	di (TA)
IL4	GAGACTTTCGGCTTTCCATTA	GCAATGTAGGAGGGTTCCA	1kb	di (TG)

represented gene	sense oligonucleotide	antisense oligonucleotide	marker distance to gene	*nucleotide marker
IL4R	TGCCAAAGGGATGTTAATTCT	CAGGACCTGACATAGCTGCT	30kb	di (GT)
IL6	TGACAGCAGAACACAAAATTG	CCAAGGATTTCTGTGGGAA	47kb	di (AC)
IL8	CTTTCACCTTTCCTCACCCT	CACTGGCTGGTCAGAGACA	14.8kb	di (AC)
IRF1	GGCACCTAAAATTCTAAGGGA	GCTACCTTGCTCTGCTGTGTA	15kb	di (TC)
LGALS3	GGAGTTTGAGACCAGCCTG	CTTTCCAAGAAAAAGGCATGT	45kb	di (AC)
LTB (TNFSF3)	CAATCCTGGACAACATAGTGG	CCACCTCCAGCCTTTCTTA	5kb	di (AG)
LTBR (TNFRSF3)	TGGGCAAGAAGAAAGTTCC	CAGTGTGGGATGTGTTCCTT	intragenic	di (AC)
LY64	ACCATCCTGGTATTGCACCTA	CATGGTGCGTAGTATCATCCA	intragenic	di (AC)
LY86	CCAACTGCTTCCAAGGATTC	GAGATTAGGGAAGCCTGATGA	intragenic	di (CA)
LY96	CCATGTGCTAATTATGCCACT	GCATGGGAATCGTTTGAAC	intragenic	di (GT)
MADD	AAGCCTCAGTTTCCCATTCT	AAGCCTTGATGAGGTTTGAAC	25kb	tri (CAT)
MAP2K6	TCTGTGGAAATGGCATTAAAG	TTAAATCACCCTGGTTGTGC	intragenic	di (AC)
MAP3K14	AAGTGGAGAGAAGCAAGGTTC	CTGGAGAGCCACAAACAGAC	0.4kb	di (GT)
MAP3K5	TCATCAGTGCAGCCAATGT	TTCTGAGGGCAGGAACCTA	intragenic	di (CA)
MAP4K4	TCTCTGCCGTGGTTGATATC	GTTCAAGGCTGCAGTGAGTTA	intragenic	di (TG)
MCL1	TGGCCATGAGCCTTCTATT	GATCCGAAATTGCACCACT	8kb	penta (TTTTG)
NCF1	CAGGCTGGTCTCAAAATCCT	CAGTTGTACACCACAGCACCT	12kb	di (GT)
NCF4	CAGGTGAAAATCCTGCTCTCT	AGTTGTGCTATCACCAGCTGA	intragenic	di (GT)
NFKB1	GAACCAGCTCAAATACCCATT	CTGCAAGTGCCATTATTTTGT	12kb	tetra (ATAG)
NFKB2	AGGTTGCAGTAAGCCGAGA	TTGGAGGGTTTCTATACCACC	7kb	tetra (AAAT)
NGFB	GAAATGGCTCCACTTTTGC	CTATCATCTGCCCTTGGCTAT	intragenic	di (TG)
NGFR	CACCTTACAGGTTGCAAAGTG	AGCATTAAAGTAACCCCTGGA	18kb	di (CA)
NME3	AGTGGCTCACACCCGTAAT	AGCCTCCTGAGTAGCTGAGTC	23.7kb	di (AC)
No1	CCCTGTGGATGTCAAGAATCT	GAGACTACCGATCCCCGAC	Chro6 marker	di (GT)
No4	GTGGACAAGGTAGGAGGCTG	AATGTTTGCCAGGAACTGTG	Chro6 marker	di (AG)
No5	AAGGAAGTCTCAAAGGCTCTG	GGTATGCCACTGCACACAG	Chro6 marker	di (TG)
No6	CAGTTTGGGCAGGACAAAG	GACAGAGCCACTGGAGAAGA	Chro6 marker	di (CA)
No7	GTTGCACTGAGCCGAGATC	TCTGTTCCAGGCCTTTCTACT	Chro6 marker	di (AC)
No8	GGAAGTGAAGACGGATCCA	GATAGCCCTGGGACATGGT	Chro6 marker	di (TG)
NOL3	CTTGACTCTCAAGGCCTGAA	CACTCCTTCTTGGAAGGCA	0.5kb	di (GT)
NOS1	ACTAAGCATTGGTGCCTGAG	GCCTGGGCAAGATATCAAG	intragenic	di (AG)
NOS2A	GGCTCCTATGGGAACATGA	CCACAGTTAACATTCGGGTG	intragenic	di (CA)
NOS3	GAACCTCTGCCCAACACAC	TCTCTTAGCATCTCCCTTTCC	intragenic	di (AC)
NOX1	CAAAACCTGCGTGTCTGATAC	CCATCCTACTCCCCAGAGATA	intragenic	di (TG)
NOX4	TCCTGTCATGGGTATTGATCC	TGCATCACACCCCACAGTA	intragenic	di (AC)

represented gene	sense oligonucleotide	antisense oligonucleotide	marker distance to gene	*nucleotide marker
NRG1B/NRG1	TCCTTCATCCCAAAGATCTCA	GCTCATGTTCTCAGAAGCCTT	intragenic	di (CA)
NSMAF	ATGCAGTGTGAGCTTTCAAAC	CCAAAAATGCGACAATGTAAG	intragenic	di (AC)
P2RX1	TGGAGATTCAGGGTAACGTG	TGATACATCTGGCCACAGGT	intragenic	di (GT)
P53AIP1	AACCAGGTAAGTGCCGTGT	CCAGCCCGTTGTCTTTAGTA	39kb	di (GT)
PAK1B	GTTCAAGCCAGTTTCACCTCT	TTGAGGGACCCTTAAGATCAT	intragenic	di (GT)
PAWR	AGAAGAATCATGCTCCCTCC	ATTTCTAATGGGATGTGTGCA	intragenic	di (CA)
PDCD10	CAGCAGTCAGCACACAGGTA	GCATGGTACTCATTGTGGAGA	14kb	di (GT)
PDCD2	CAACGGAATAAAATAGGGCTT	ACCGACTTCTCTCAGGCTTC	50kb	di (GT)
PDCD5	GTTGCGGTGAACCAAGATT	GTGACAGAGCACGATCATGA	39kb	di (AC)
PDCD6IP	CATGCCTGTAGTCCCAGCT	ATGTGCTGATTGACCATTTGT	27kb	tetra (AAAT)
PDCD8	CCAGACCCTGTCTCTCAAAAT	CTCACCCCCACTAGTTCCA	intragenic	di (GT)
PGLYRP	GGCTGTTTAAAATAGGCCACA	TCCAAGTGGCAGAACAGGT	8.7kb	di (GT)
PIAS3	CCAGCCTGAGTGACAGAGTG	CCCTGGCAATAGGAAACTTC	6kb	tetra (tatc)
PLA2G10	AGTGCATAGTCTGGCTTCCTT	ACTTTGGGTCGAAGCAGTAAT	intragenic	di (AC)
PLA2G1B	GTGCCAGGCTCTGTCCTAG	GGTTGTAAGCTCCATGAGGTT	intragenic	tri (ATA)
PLA2G4A	ACCTCCATTCCATCTCCTTTC	CAGAACGAGGAATGTGGGA	intragenic	di (GT)
PLA2G6	TCCCTGCTGGAATCAGAAG	CTGGGTTACAGAGCAAGACCT	intragenic	di (AC)
PLUNC	AGTCCCACAGGTCTTGCAG	CTGGAAATGGGTGTAAGGGT	intragenic	di (TG)
PRL	CAGCTGTGCTGTTTGGAGTC	ACCCCAGAATTATTCTGGACA	14kb	di (AC)
PRLR	TGAGGGTAAGGCAATACACAA	ATGGGCTTGACAAAGTGGT	intagenic	di (AC)
PTEN	GGCATTTGGCTTAGTTCAAGT	TGTTTCAGAACTCTCCTGCTG	intagenic	di (TG)
PTGS1	CTTGTGGTCCTTGCTTGTGT	AGTCCTCTTGGTCCCCACT	intragenic	di (TG)
RARB	CCCCTTTTCCAGCTGTATCT	AGGAGTAAGAAAGTGCAGGGA	intragenic	di (CA)
Rb2/p130	AGGCAAGAGAATCGCTTGA	TGGGCAACAAGAGTGAAACT	22kb	di (TG)
RbAp48	CCATGCTTTTGTATGAGCTGT	GGTTGCAGTGAGCCAAGAT	7kb	tri (AAT)
RBP1	CTTCCATGGGAATCTCTGG	TGCTCACTCTTCCCTGGTT	intragenic	di (TA)
RBP2	TGGGAGGGAGATGTAGCAC	AGCAAACTCTTAGGAGGATGC	intragenic	di (AC)
RBQ-1	TGAGGCCCCACATACTTGT	GCTGGTCTCAAACTCCTGACT	14kb	di (GT)
RBQ-3	TGCATCTTGGGTGACA	TGACAGGGTTTTACCATGTTG	59.5kb	di (AC)
REQ	GACCTTGGAATCCCAGACA	GAGGCGGAGGTTGTAGTGA	9kb	di (GT)
RIPK1	GCCTGGGTGACAAGAGTGT	CAGATCCACCAGAGGAATCA	3kb	tri (AAT)
RIPK2	ACATCAGCAAGAAGTCACGAA	CTGAAATCCTCGGCACACT	11kb	di (GT)
RIPK3	CCCTGACTCTCTAGACTCCCA	AGTTGTGGTGAGCCGAGAT	10kb	di (GT)
RIPK5	TGCATCTTGGGTGACA	TGACAGGGTTTTACCATGTTG	intragenic	di (AC)
RNF7	ACAATGCTTGACACGATGC	GGCAGAGTATTTGATCAGGCT	17kb	di (GT)

represented gene	sense oligonucleotide	antisense oligonucleotide	marker distance to gene	*nucleotide marker
RXRB	AAGGTAGGAGGCTGTGGGT	AATGTTTGCCAGGAACTGTG	5kb	di (AG)
SerpinA1	ACAGGAAGCTAATCAAGGCAT	GTGACAGTGCAAGACTCCATC	45kb	di (TG)
SerpinB1	ATTCTTTTCTCCCCATGTCAG	AGTGGAACAGAGGTCACCCT	28kb	di (TG)
SFTPA1	AAAACCAAGAAGAGGGCAAT	TGGACATTTTCACCCCTCTA	19.1kb	di (GT)
SLPI	AGTCGCAGACAGGTGAAGAA	GCTCCAAGGTTTGTGTGACA	intragenic	di (GA)
SMAC	CTGGGCAACAAGAGTGAAAC	CCTCCAAATGTTAAATGCAGA	33kb	tetra (ATAC)
STAT3	AGCTTTGCCCTCCTAAGAGA	GAGTCTGGAGTCCACGTTCA	intragenic	di (AG)
STK17A	GTATTTGGCATTCCTTGGC	AGGTCATTAGCGTTAGCCCTA	15kb	di (GT)
STK17B	GACAGAGCAAGACTCCAACAA	GGTGGCAATAAAGTCAGGTTT	intragenic	tri (CAA)
TANK	GGAGCTCAGATTCCATTGTG	GGAATGCACTCTAAGCCAATT	intragenic	di (AC)
TAPBPR	CAAGCTGGAACTTCCTGAGA	GGGAACAGAGCAAGATTCTGT	intragenic	tetra (TTTA)
TGFB1	CTTTTGTCCACCCTGCTGT	CTATGATCGCTCCACTGCA	24.9kb	di (ga)
TIAF1	AGAACAATCTGCTGGTTTCG	GTCAAGAGGAATGGCTCCA	intragenic	di (GT)
TIAL1	CATGCACGGACAAACATACA	GGATATTCGAAGCAAGGGTAA	21kb	di (AC)
TLR1	CTGCTTGTTTATCCCATTCG	TCTCAGTGGAAGAAGAGGGTT	intragenic	di (GT)
TLR10	CCCACAATAGACCTTGCAATA	GCTCTCGACCTAGAAAAGCAT	1.4kb	di (TA)
TLR2	GACTGGCATACCACAAAGTTG	CTGGGCAAGACAGAGTTGAG	10kb	di (AT)
TLR3	GGAAGGGTATGCAGCTGTTC	GTGAGTCAAGATCGTGCCAT	16.5kb	tri (tta)
TLR4	AGAAGTGCCCTCAAAGTTACG	CCTGCATTTTGGCATAACA	8kb	di (AC)
TLR5	TCGCTTATTTTTGGCAACTT	CCAGTGGGTGGCTGTAGTACT	17.7kb	di (GT)
TLR7	CCTTTTCCTTGTTTGTTTGGT	AACCTGCATGTTGTGCACA	32.2kb	di (TA)
TLR8	CATGAGGTGGATGTTGCAG	GTGGCCATTTTTTGCAAGT	intragenic	tri (AAC)
TLR9	ACAGAGTGAGACGCCGTCT	GATGAGCACAATGCCATTCT	32kb	tetra (AAAG)
TNFa	GCCTCTAGATTTCATCCAGCCACC	CCTCTCTCCCCTGCAACACACA	7.8kb	di (AC)
TNFRSF10A	CTGTCATCTGGTGCAGACCT	TCTCTGCAGTGTCCTTTGGT	25kb	di (GT)
TNFRSF10B (SCAM1)	ACAAATCCAGGAGTCAGAGGA	AAGAGCTACTGACCCACTGCT	intragenic	di (GT)
TNFRSF10C	TGAATACATCTTTGGCCACAT	GAAATGCATTTGGCGTCA	9kb	di (CA)
TNFRSF10D	TGGGTGACAGAGCAAGACTC	TTCACATCCTTGGGTAGAGGT	intragenic	di (AC)
TNFRSF11A	TCCTTAGCTGTGCTTGTTCTG	TCTGGGATACTGGCAAGAGA	intragenic	di (AC)
TNFRSF11B	AGTAGCTTGGAAGTTCCATGC	GTACCACTGCACTCCAGTCTG	intragenic	di (GT)
TNFRSF12	AGCTGAAGTGCCACTGTCC	AACCCATTCTGTCCCATGTAC	3kb	tri (TTC)
TNFRSF17	GGATTCCCAAGCATGTACAG	ACAGAGCGAGACTCCATCTCT	intragenic	tetra (ATTT)
TNFRSF18	TTCAGCCTCTGGTATTCCTTT	CCTTCTATCCATGGCCTCA	30kb	di (AC)
TNFRSF19	CTTTGTTTGCCCGTTACCTAT	AACCAGGAGTCAAAGTGGAAG	intragenic	di (GT)
TNFRSF19L	ACTCCGGACTGTGTTTTGAA	CCTGTTCTCATGGCATGTAAC	3.5kb	tri (TTA)

represented gene	sense oligonucleotide	antisense oligonucleotide	marker distance to gene	*nucleotide marker
TNFRSF1A	ACTCTTGTCATCCCTGGACAT	CTTCCCTCTTCCCATTCACT	20kb	di (AC)
TNFRSF1B	AGGGAAGCCATAAATCCAGA	TGCCTGTAACAGGGACAAGT	intragenic	tri (ACC)
TNFRSF21	CTCAAGCACTGGCCATATTC	CTCAATTTAGTGGCAGGGTG	intragenic	di (AC)
TNFRSF4	TTCAGCCTCTGGTATTCCTTT	CCTTCTATCCATGGCCTCA	50kb	di (AC)
TNFRSF5	GATCTTCCTGTGCCCATATG	CTTCCACCTTTGCTGTTGAG	35kb	di (GT)
TNFRSF6	GGTCTGGGATAAAATTCAGGA	GGTACTGTGTCACCCACTCC	26kb	di (CT)
TNFRSF6B	AGCAAGACTCTACTGCGCAC	CACACCCACACCATCATTAGT	34kb	di (AC)
TNFRSF7	CAAGCTGGAACTTCCTGAGA	GGGAACAGAGCAAGATTCTGT	8kb	tetra (TTTA)
TNFRSF8	AGGGAAGCCATAAATCCAGA	TGCCTGTAACAGGGACAAGT	61kb	tri (ACC)
TNFRSF9	GCTGCCAGTTGTGTAAAGTGT	ACAGGTGTGAGCCACAACA	intragenic	di (AT)
TNFSF10	GGGAGCCTGTTATAAATGCA	GAGCTGAGATCGCACCACT	19kb	tri (TTA)
TNFSF11	ATTACAGGCATGAGCCACC	CCTGAGGAGAAAAGGAGCAT	12kb	di (GT)
TNFSF12	CCCATGCATTTTTAATTCCA	GCAACATAGTGAGACCGCA	112kb	di (GT)
TNFSF14	CCCTATGAAGAGACTGCCCT	CCTGTGTCATTGTGGGTCA	intragenic	di (AT)
TNFSF15	CCACTACACCCAACCCATT	AAGCAACCAATAAGGCTGC	40kb	di (GT)
TNFSF18	GTGGCATGTTGTTTTGTGTG	GCAAGTCATTCTTCCCATCA	4kb	di (GT)
TNFSF4	AATGCCCATCAATCAACAAG	TTTCATCCAGATTGCTGCA	12kb	di (AC)
TNFSF5	GAGCTGAGATCGTGCCATT	GTGTGCCAAGGCTAATTCTCT	3.4kb	tetra (CAAA)
TNFSF6	ACTGGCAGCATCTTCACTTC	TTCAAAATCTTGACCAAATGC	intragenic	di (GT)
TNFSF7	ACACAAAGATAAAGGCTGGGT	TGGACTCTCAGATGCATATGC	8kb	di (GT)
TNFSF8	GCTACAGAGAAGGTGGCTGAT	TGGCAGAGTCTAAACGAAACA	0.05kb	di (TG)
TNFSF9	ACACAAAGATAAAGGCTGGGT	TGGACTCTCAGATGCATATGC	50kb	di (GT)
TOSO	CCATCTGCTGAAGCCTAGAA	ATGCAGTGTGAGCTTTCAAAC	intragenic	di (GT)
TP53	TGGGCGATAGAACAAGACTC	ATTCTGGTCCCTGTGTTCCT	23kb	tri (AAT)
TP53INP1	ACCCATCAAATTCACAGCAC	GCCAACATAAATCTGTCCCA	intragenic	di (TG)
TP73	TTATCTGAACTTGCTGCACGT	GTCAAACCATGAAATTGCTGA	intragenic	di (GT)
TRADD	CTTGACTCTCAAGGCCTGAA	GTTGGACAGGACACTCCTTCT	16kb	di (GT)
Traf1	TGCAGCAGAAATGAACCTG	TCTTGGGTGGCTGGTATATG	12kb	di (GT)
Traf3	TGGCACCACTATACTCCAGC	GAAATGCCAAATTGTGCCT	intragenic	di (AC)
Traf4	TTAACAGAGCCCCATTCTTG	GAGGTGAGTTCTTGCCACTG	58kb	tri (TTA)
Traf5	ACTTGGCCAGCACATTATACA	GTCAAGGAGCATCTGTGCAT	intragenic	di (AC)
Traf6	GGTCTTGGTTTGTCCAGATTT	TTGAAAACAGTACAGGCATCC	68kb	di (GT)
TrkC	CAACTCAAGGCCCTTCCTA	GCTCTCCTTCACTGCCTACA	intragenic	di (GT)
VDR	CAGGAGTTTGAGTCTGCAGTG	CATCCCCCTCATTCTGTACTT	5kb	di (AC)
VEGF	GTGAATGCACTTGGAGAAAGA	GCAGTCTCCCCTAAACTCCTA	13kb	di (AC)

Information of individually genotyped microsatellite markers with significant differences in the allele distribution

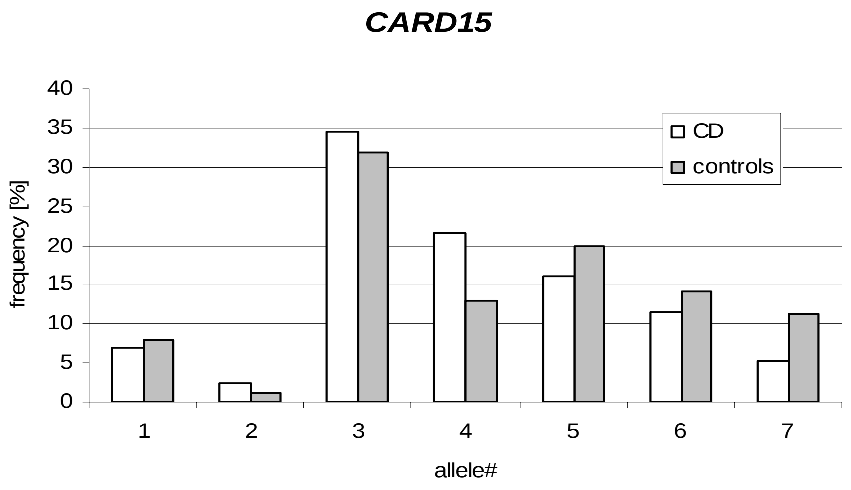
CARD15
frequency [%]
40
35
30
25
20
15
10
5
0
1
2
3
4
5
6
7
allele#
CD
controls

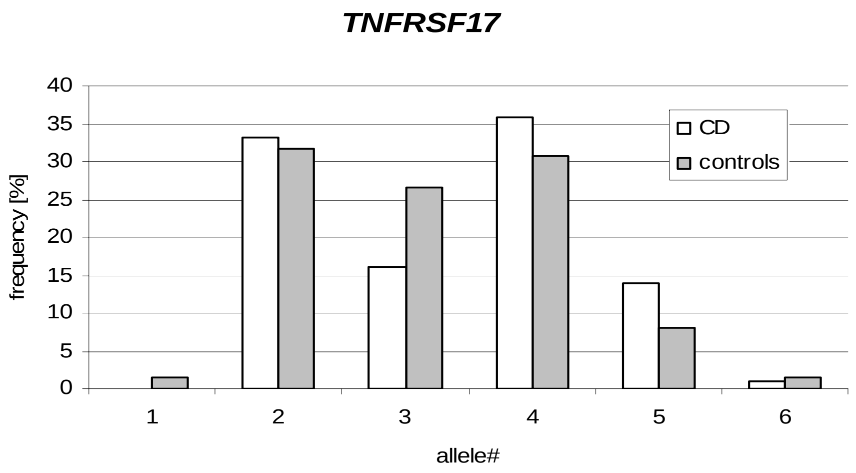
TNFRSF17
frequency [%]
40
35
30
25
20
15
10
5
0
1
2
3
4
5
6
allele#
CD
controls

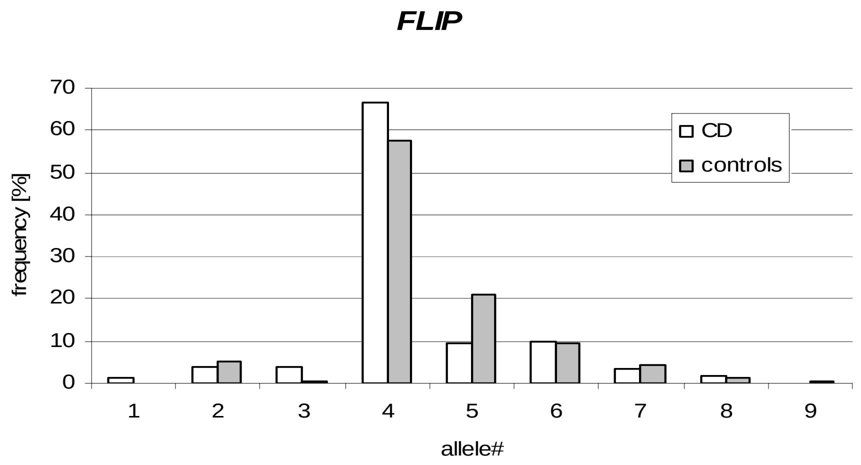
FLIP
frequency [%]
70
60
50
40
30
20
10
0
1
2
3
4
5
6
7
8
9
allele#
CD
controls
